# Spatial Distribution of Bedding Attributes in an Open Compost-Bedded Pack Barn System with Positive Pressure Ventilation in Brazilian Winter Conditions

**DOI:** 10.3390/ani13050786

**Published:** 2023-02-21

**Authors:** Carlos Eduardo Alves Oliveira, Ilda de Fátima Ferreira Tinôco, Victor Crespo de Oliveira, Pedro Henrique de Moura Rodrigues, Leonardo França da Silva, Flávio Alves Damasceno, Rafaella Resende Andrade, Fernanda Campos de Sousa, Matteo Barbari, Gianluca Bambi

**Affiliations:** 1 Department of Agricultural Engineering, Federal University of Viçosa (UFV), Viçosa 36570-900, MG, Brazil; 2 Department of Engineering, Federal University of Lavras (UFLA), Lavras 37200-900, MG, Brazil; 3 Department of Agriculture, Food, Environment and Forestry, University of Firenze, 50145 Firenze, Italy

**Keywords:** dairy cattle, housing systems, composting, bed quality, geostatistics

## Abstract

**Simple Summary:**

The use of compost-bedded pack barn (CBP) systems for dairy cattle housing can improve animal welfare and herd productivity, but it is necessary that the bedding is properly managed. Therefore, evaluating the bedding quality conditions in this confinement system is paramount, as the results obtained can be used as a basis for adequate management. In this study, bedding attributes were mapped in a CBP with positive pressure ventilation. From the mapping, it was possible to identify areas with unsuitable conditions for the staying of the cows (surface) and for the composting process (at 0.2 m depth). The results achieved in this study can be used to guide decision-making processes regarding bedding management in this housing system.

**Abstract:**

The objective of this study was to characterize the dependence and spatial distribution of bedding attributes in an open compost-bedded pack barn (CBP) system with positive pressure ventilation during the winter period in Brazil. The study was conducted in July 2021, in the Zona da Mata region, Minas Gerais, Brazil. The bedding area (shavings and wood sawdust) was divided into a mesh with 44 equidistant points. At each point, the bedding temperature at the surface (t_B-sur_) and at a depth of 0.2 m (t_B-20_) and the air velocity at bedding level (v_air,B_) were measured, and bedding samples were collected. The bedding samples were used to determine the moisture level and pH at the surface (M_B-sur_ e pH_B-sur_) and at a depth of 0.2 m (M_B-20_ and pH_B-20_). The spatial behavior of the variables was evaluated using geostatistics techniques. For all variables, the occurrence of strong spatial dependence was verified. Through the maps, it was observed that t_B-sur_, t_B-20_, M_B-sur_, M_B-20_, and v_air,B_ showed high spatial variability, whereas pH_B-sur_ and pH_B-20_ demonstrated low variation. On the surface, values of t_B-sur_ < 20 °C and M_B-sur_ > 60% were observed. At the subsurface, there was a predominance of t_B-20_ < 40 °C, M_B-20_ > 60%, and pH > 9, which are indications of low bedding composting activity.

## 1. Introduction

The use of facilities free of stalls for dairy cattle housing, able to improve the thermal comfort conditions and ensure greater freedom of movement for the animals, has grown in Brazil in recent decades [1,2]. A system that is becoming prevalent is the compost-bedded pack barn (CBP), which, in addition to enabling improvements in the comfort, productivity, health, and longevity of the herd, has a lower cost of implementation and greater environmental appeal, as it uses less water to carry waste and enables the formation of compost with good agronomic features [1,3,4,5,6].

In CBPs, the cows are housed in facilities with a large covered area, with bedding composed of a soft and comfortable organic material substrate, which, under certain conditions, undergoes the decomposition process over time [7,8]. In this system, the bedding management must ensure hygienic and comfortable surface conditions for the animals, at the same time as enabling the provision of subsurface conditions conducive to the development of aerobic decomposing microorganisms [1,7,9,10]. To promote improvements in the bedding drying process, enhance the activity of aerobic decomposing microorganisms, and carry out the incorporation of waste, the material must be revolved and aerated daily, two to three times [4,11].

Bedding composting in CBP systems is a biological process, influenced by factors such as temperature, humidity, hydrogenionic potential (pH), nutrient concentrations, and aeration of the bedding material [1,12]. Thus, variables such as temperature, humidity, bedding pH, and air velocity at the bedding level must be monitored and evaluated frequently. These parameters can be used as indicators of the thermal and hygiene conditions, when evaluated on the bed surface, and of the development of the composting process, when evaluated in the aerobically active layer (0.15–0.30 m) [7,12].

In fact, evaluating and monitoring the bed environment in CBP systems is important, as the results obtained can be used as a basis for adapting the management applied to the bedding, considered a key point for this system’s success [1,4,10]. However, few works with this objective were carried out in Brazilian climatic conditions, and this is a knowledge gap that has not yet been solved.

For bedding evaluation and monitoring, innovative computational methods should be used, such as geostatistics, a tool that makes it possible to evaluate dependence and spatial distribution and interpret the results from the natural structuring of the data [13,14]. The use of this tool has been shown to be satisfactory in mapping the thermal and the bedding environment in CBP systems, making it possible to analyze the spatial distribution of the parameters of interest more precisely [5,15,16,17,18,19,20,21]. In view of the above, the objective of this study was to evaluate and characterize whether the variables temperature, humidity, and bedding pH, as well as air velocity at the bedding level, have dependence and spatial variability in relation to the internal area of one compost-bedded pack barn system with positive pressure ventilation, during the winter period in Brazil.

## 2. Materials and Methods

This study was conducted during three consecutive weeks, in July 2021, the winter period in Brazil. At this time, extreme conditions are observed regarding the management of bedding material in compost-bedded pack barn (CBP) systems, due to excessive humidity and low temperatures. These conditions can compromise the quality of the composting process, cause increased animal soiling, and lead to a loss in milk quality.

The study was approved by the Ethics Committee on Animal Use of the Federal University of Viçosa (Process 04/2021). All procedures described were conducted in accordance with the guidelines recommended by the committee.

### 2.1. Characterization of the Compost-Bedded Pack Barn Installation and Management Techniques

To conduct this study, experimental data were collected in a confinement facility for lactating dairy cattle, in an open CBP system with positive pressure ventilation. The facility at which the study was conducted belongs to a commercial property located in the mesoregion of Zona da Mata, Minas Gerais, Brazil (latitude 20°46′41″ S; longitude 42°48′51″ W; and altitude 670 m). According to the Köppen climate classification, the climate in the region is Cwa: Mesothermal subtropical, with cold winters and hot and rainy summers [22].

**Figure 1:**
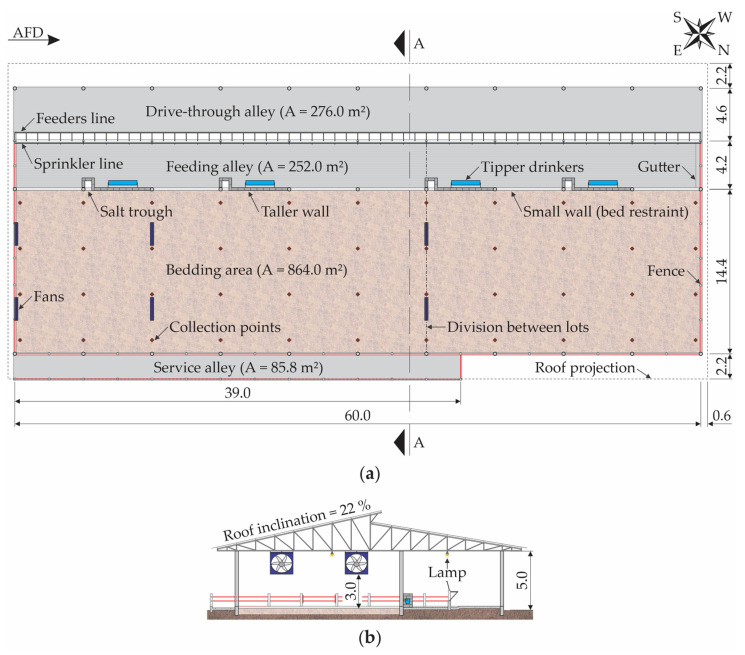
Schematic representation of the compost-bedded pack barn system where the data were collected: floor plan with collection points (**a**) and cross-sectional AA (**b**). AFD—air-flow direction; dimensions in meters (m); Source: Oliveira et al. [19].

The barn was built in July 2019, was oriented in the southeast–northwest direction and had the following construction characteristics: 60.0 m length; 27.6 m width; 5.0 m of eave height, gabled roof, with structure and metal roof tiles; a central opening with 1.0 m overlap and 0.8 m eaves (Figure 1). The feeding alley was 4.2 m wide, located in the southwest region of the facility, and had four tipper drinkers (2.0 m length each) and a single feeder (60.0 m length).

The facility had positive pressure ventilation, provided through six mechanical fans with low volume and high rotation (LVHS). The fans were installed along the facility length, specifically two three-propeller fans, with 1.52 m diameter, 1.5 hp and 86,000 m^3^·h^−1^ air flow, installed on the southeast face of the facility, and four six-propeller fans, with 1.53 m diameter, 2.0 hp and 55,000 m^3^·h^−1^ air flow, installed in pairs (levels 12.0 and 36.0 m, in relation to the southeast face of the facility, Figure 1). Such equipment was installed at a 3.0 m height, with a 45° inclination, and remained activated 24 h·day^−1^.

The facility had a lighting system consisting of 100 W LED lamps (nine in the central region of the bedding and nine in the border region between the feeding alley and drive-through alley). The lighting system was activated only at night (06:00 p.m. to 06:00 a.m.).

On the sides, as well as between the bedding area and the feeding alley, there was a small 0.2 m-high wall, with the functions of preventing bedding material loss, passage of bedding material to the feeding alley, and/or waste for the bedding area. In places where tipper drinkers were installed, taller walls (1.2 m) were built to contain the animals, preventing them from having access to water directly from the bedding area, which could cause wetting. In the feeding, service, and drive-through alleys, the floor was composed of beaded concrete.

In the evaluated system, the “bedding” substrate consisted of a mixture of wood shavings (<8.0 mm) and sawdust (8.0–25.0 mm) from *Eucalyptus*, with a thickness of approximately 0.6 m and use time of four months (at the beginning of the study). For the bedding composition, a 0.3-m-thick dry sawdust was added, which, together with the waste deposited by the animals (feces and urine), started the composting process. Subsequently, dry materials (wood shavings and sawdust from *Eucalyptus*, with moisture content between 10 and 15%) were added whenever the bedding moisture was too high, causing increased dirtiness among animals, excessive compaction, and a consequent anaerobiosis situation in the compost. During the entire experimental period, only bedding replacement with dry material was performed (twice, approximately 8 tonnes each, when it was observed that the bedding was excessively wet).

To rotate the bedding material, a hybrid device was used (bedding rototiller with cultivator, 2.0 m wide, five rods, 540 rpm rotational speed of the working element, 0.5 m maximum depth and 0.3 m of effective depth), coupled to a tractor (light line, 78 hp and 2400 rpm nominal rotation of the engine). Turning was conducted twice daily (09:00 a.m. and 04:00 p.m.), according to the farm’s standard routine.

During the experimental period, the animals housed inside the facility were distributed in two pens (established by the farm), according to milk productivity. The animals with the highest productivity were housed in Pen 1, which had a 518.4 m^2^ bedding area (36.0 × 14.4 m) and was located near the southeast face of the facility. The animals with lower productivity remained housed in Pen 2, which had a 345.6 m^2^ bedding area (24.0 × 14.4 m) and was located near the northwest face of the facility. During the experimental period, 80 Holstein cows (pure of origin, 600 kg average weight, in lactation) were housed inside the facility [45 in Pen 1 (11.52 m^2^·animal^−1^) and 35 in Pen 2 (9.87 m^2^·animal^−1^)]. The area available per cow met the welfare standards recommended by the Ethics Committee on Animal Use of the Federal University of Viçosa.

The standard routine of activities in the system was maintained throughout the experimental period, with two milkings being performed and roughage provided twice daily. Milking started at 04:00 a.m. and at 04:00 p.m., with a 2 h 30 min average duration, and were performed in a 2 × 6 fishbone-type room, located in a facility attached to the CBP system, where a waiting area was also located. At all times, the cows had access to the feeding alley, where food (total mixed ration) and water were provided, both ad libitum. The feeding alley floor was washed once a day, in the morning, using a flushing system. For the cows to become familiar with the experiment presence, the experimental collections were performed only from the third experimental day.

### 2.2. Characterization of the Thermal Microenvironment

To characterize the thermal microenvironment, data on the dry-bulb air temperature (tdb) and relative humidity (RH) inside the facility were collected every 5 min, 24 h·day^−1^, throughout the experimental period. The thermal environment data were used as background for consecutive inferences about the bedding attribute results.

In the bedding facility area, the tdb and RH collections were performed using DHT22 sensors (model AM2302; temperature measurement range from −40 to 80 °C and accuracy of 0.5 °C; humidity measurement range from 0 to 100% and 2% accuracy; Aosong Electronics Co., Ltd., Guangzhou, China). The data collected by the sensors were processed and stored using collection stations, which consisted of an Arduino Uno R3 board (ATmega328 microcontroller; 5.0 V supply voltage; 16 MHz clock speed; Atmel Corporation, San Jose, CA, USA), connected to a Data Logger Shield with RTC and SD Reader (SD card slot, integrated real-time clock DS1307; FAT16 or FAT32 card formatting; 3.3 V supply voltage; Dallas Semiconductor, Dallas, TX, USA) and an LCD Display 16 × 2 (I2C Backlight Blue, 5.0 V supply voltage; 4 or 8 bits communication; Beijing Qingyuan Innovation and Technology Development Co., Ltd., Shenzhen, China), following a methodology adapted from Freitas et al. [23]. The sensors were installed 2.5 m above the bedding level, to allow the passage of the tractor used for bedding turning.

To characterize the air currents available for the drying of the bedding surface layer, air velocity data were collected at the bedding level (v_air,B_). The collections were always carried out in the morning (09:00 a.m.), twice in each experimental week, constituting six repetitions. Data were collected using a hot wire anemometer (model TAFR-190, with a scale between 0.1 and 25.0 m·s^−1^ and 5% accuracy, Instrutherm Instrumentos de Medição Ltda., São Paulo, SP, Brazil). The collections were always carried out with the animal’s presence.

To collect the tdb, RH, and vair,B data, the bedding area was divided into a regular mesh (6.0 × 4.5 m), consisting of 44 equidistant points, distributed according to the system’s construction characteristics (Figure 1a). Data collection for the characterization of bedding attributes was performed using the same mesh.

### 2.3. Characterization of Bedding Material

The characterization of the bedding material present in the system was performed by determining the variables of temperature, humidity, and bedding hydrogenionic potential (pH), on the surface and at a depth of 0.2 m. The bedding temperature collection and bedding material samples for the determination of moisture and pH were carried out during the three experimental weeks, twice a week, constituting six repetitions. Data and bedding samples were collected immediately before turning over the bed (08:00 a.m. to 09:30 a.m.), during which time the bed remained for between 16 and 18 h without being turned over. At each mesh point (Figure 1a), bedding temperature data and bedding samples were collected at the surface and at a depth of 0.2 m.

For data collection on the bedding surface temperature (t_B-sur_), a digital infrared thermometer was used (model 62 MAX, with a measurement capacity of between –30.0 and 500.0 °C and 1.5% accuracy, Fluke Corporation, Everett, Washington, WA, USA), with a 1.0 m focal length and with emissivity (Ɛ) adjusted to 0.9, as recommended by Silva et al. [16]. For bedding temperature measurements at a depth of 0.2 m (t_B-20_), a rod thermometer was used (model TP101, with a scale of −50.0 to 300.0 °C and 2% accuracy, Pyromed Instrumentos de Medição e Controle, Contagem, MG, Brazil), which was inserted into the bed for approximately one minute at each collection point, or until its temperature reading stabilized.

To determine the moisture and pH values of the bedding material, samples were collected at each of the sampling points. The collections were carried out on the surface and at a depth of 0.2 m, using a post hole digger with marked depth values. The material was collected in the morning, immediately before turning over the bed, twice a week, during the three experimental weeks. The samples collected at each point were placed in hermetically sealed and duly identified plastic packages. After each collection, the samples were placed in isothermal boxes and sent to the Laboratory of Anaerobic Digestion of the Center for Research in Ambience and Engineering of Agroindustrial Systems at the Federal University of Viçosa, in Viçosa, Minas Gerais, Brazil.

The pH of the bedding samples was measured in distilled water, using 10 g of the compound sample and 25 mL of distilled water (ratio 1.0:2.5), according to the methodology described by Zhao et al. [24]. To perform the readings, a digital benchtop pH meter was used (model PH-2600, with a measurement scale between 0 and 14 and 0.01 precision, Instrutherm Instrumentos de Medição Ltda., São Paulo, SP, Brazil), duly calibrated (Instrutherm Calibration Certificate Number 121239/21).

The moisture of the bedding samples was determined using the standard oven method (gravimetric), following the methodology described by Teixeira et al. [25]. Samples were weighed using an analytical balance (model AY220, with a 220.0 g measuring capacity and 0.0001 g accuracy, Shimadzu Corporation, Kyoto, Japan), duly calibrated, and drying was carried out in an oven at 105.0 ± 5.0 °C. The moisture, as a percentage, was calculated through the ratio between the mass of water removed and the mass of dry sample, multiplied by 100.

### 2.4. Statistical Analysis

#### 2.4.1. Descriptive Statistics Analysis

To characterize the thermal microenvironment inside the CBP system, the hourly mean value and standard deviation of the dry-bulb air temperature (t_db_) and relative humidity (RH) variables were calculated. From the calculated data, daily behavior curves of these variables were generated.

The variables of bedding temperature at the surface (t_B-sur_), bedding temperature at a depth of 0.2 m (t_B-20_), bedding moisture at the surface (M_B-sur_), bedding moisture at a depth of 0.2 m (M_B-20_), bedding pH at the surface (pH_B-sur_), bedding pH at a depth of 0.2 m (pH_B-20_), and air velocity at bedding level (v_air,B_) were initially analyzed using descriptive statistics, through the R Development Core Team computer system [26]. Mean, median, minimum, maximum, standard deviation (SD), and coefficient of variation (CV) data were obtained. The variability of the experimental data was evaluated using the CV classification proposed by Warrick & Nielsen [27]: CV < 0.12 = low dispersion; 0.12 ≤ CV < 0.24 = moderate dispersion; and CV ≥ 0.24 = high dispersion.

#### 2.4.2. Geostatistics Analysis

The spatial behavior of the attributes of interest (t_B-sur_, t_B-20_, M_B-sur_, M_B-20_, pH_B-sur_, pH_B-20_, and v_air,B_) was analyzed using geostatistics techniques, which made it possible to verify whether they had a dependency and visualized their spatial distribution. The analyses were performed using the R Development Core Team computer system [26], through the geoR library [28].

Using Matheron estimator [29], semivariogram adjustments were performed (Equation (1)).

(1) $$\hat{\gamma }\left(h\right)=\frac{1}{2N\left(h\right)}\overset{N\left(h\right)}{\underset{i=1}{\sum }}{\left[Z\left(X_{i}\right)-Z\left(X_{i}+h\right)\right]}^{2}$$

where $\hat{\gamma }\left(h\right)$ is the semivariance, $N\left(h\right)$ is the number of pairs of experimental observations $Z\left(X_{i}\right)$ and $Z\left(X_{i}+h\right)$, and h is the distance between the experimental observations.

The experimental semivariograms were fitted using the ordinary least squares (OLS) and restricted maximum likelihood (REML) methods. The spherical, exponential, and gaussian models (Equations (2)–(4), respectively) were tested, as described by Vieira et al. [30].

(2) $$\hat{\gamma }\left(h\right)=C_{0}+C_{1}\times \left[1.5\times \left(\frac{h}{a}\right)-0.5\times {\left(\frac{h}{a}\right)}^{3}\right],\;\mathrm{if}\;h\leq a\;\mathrm{or}\;\hat{\gamma }\left(h\right)=C_{0}+C_{1},\;\mathrm{se}\;h>a$$

(3) $$\hat{\gamma }\left(h\right)=C_{0}+C_{1}\times \left[1-e^{\left(\frac{-3h}{a}\right)}\right]$$

(4) $$\hat{\gamma }\left(h\right)=C_{0}+C_{1}\times \left\{1-e^{\left[-3\times {\left(\frac{h}{a}\right)}^{2}\right]}\right\}$$

where $C_{0}$ is the nugget effect, $C_{1}$ is the contribution, and a is the range.

The evaluation and choice of the adjusted semivariograms was carried out using cross-validation procedures, calculating the mean error (ME), mean-error standard deviation (SD_M_), reduced error (RE), and reduced-error standard deviation (SD_R_), as detailed by Ferraz et al. [31]. For each variable, the semivariogram adjustment was chosen in which the ME and RE were closer to zero, lower SD_M_ and SD_R_ closer to one, as recommended by Isaaks and Srivastava [32]. From the mathematical models $\hat{\gamma }\left(h\right)$ chosen, the coefficients of the theoretical semivariogram were obtained, referred to as the nugget effect ($C_{0}$), contribution ($C_{1}$), sill ($C_{0}$ + $C_{1}$), range (a), and practical range ($a^{\prime }$).

For spatial dependence analysis, the spatial dependence index (SDI) was used, obtained through the ratio between the nugget effect ($C_{0}$) and the sill ($C_{0}$ + $C_{1}$). The SDI assessment was performed using the classification by Cambardella et al. [33]: SDI ≤ 0.25 = strong spatial dependence; 0.25 < SDI ≤ 0.75 = moderate spatial dependence; and SDI > 0.75 = weak spatial dependence.

Once the occurrence of spatial dependence was verified, interpolation was performed using ordinary kriging to obtain the levels of the variables in non-sampled locations in the bedding area. Based on the interpolated data, response surface maps were generated using the computational program ArqGIS^®^, version 10.1, licensed for use by the Department of Agricultural Engineering of the Federal University of Viçosa (DEA/UFV). Seeking to obtain greater detail in the variables’ spatial distribution, the percentages of area occupied by each interval were calculated and histograms generated.

## 3. Results and Discussion

### 3.1. Hourly Average Characterization of the Thermal Microenvironment Inside the Facility

**Figure 2:**
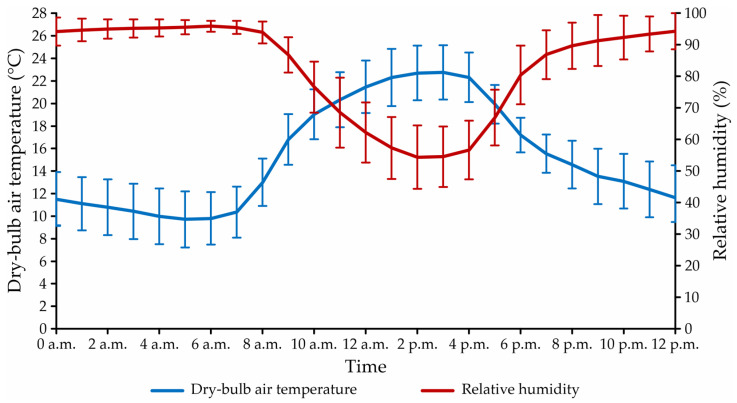
Curves of average hourly values, with standard deviation, of dry-bulb air temperature, and relative humidity throughout the day inside the CBP facility.

Figure 2 shows the hourly curves of the average data recorded during the winter experimental period for dry-bulb air temperature (t_db_, in °C) and relative humidity (RH, in %), inside the open compost-bedded pack barn facility.

As seen in Figure 2, the average values of t_db_ inside the facility ranged from 9.7 ± 2.5 °C (05:00 a.m.) to 22.8 ± 2.4 °C (03:00 p.m.). The lowest mean t_db_ values were recorded in the morning (00:00 a.m. to 06:00 a.m.) and night (06:00 p.m. to 00:00 a.m.) periods, when the mean t_db_ was less than 18.0 °C. The highest mean values were recorded in the afternoon period (12:00 a.m. to 6:00 p.m.), when the mean t_db_ was greater than 18.0 °C.

For RH, it was observed that the hourly average values curve had behavior contrary to that observed for t_db_, with the occurrence of high values in the night and dawn periods (06:00 p.m. to 06:00 a.m., RH > 80.0%) and decreasing values in the morning and afternoon periods (06:00 a.m. to 06:00 p.m., RH ≤ 80.0%). Even during the day, the mean RH values inside the facility remained high (≥60.0%). Further details on the thermal variations during the experimental period within the evaluated CBP system can be found in Oliveira et al. [19].

### 3.2. Characterization of the Spatial Variability of Bedding Attributes

Table 1 lists the methods, models, and parameters estimated from the adjusted experimental semivariograms for the variables t_B-sur_, t_B-20_, M_B-sur_, M_B-20_, pH_B-sur_, pH_B-20_, and v_air,B_. For all variables, the best adjustments were obtained using the restricted maximum likelihood (REML) method (Table 1). This method has been used to adjust small data groups, such as those commonly found in the animal ambience area, as it results in less biased estimates [34,35].

For the variables M_B-sur_, M_B-20_, pH_B-sur_, and pH_B-20_, the best fits of experimental semivariograms were obtained using the spherical model, while, for t_B-sur_, t_B-20_, and v_air,B_ the Gaussian model returned better fits (Table 1). Both models are adequate to describe the spatial variability of these variables, since their functions are conditional positive, a condition that ensures that the calculated variances are also positive [30,36]. The adequacy of the cited models to describe the spatial variability of the variables was reinforced by the data obtained by cross-validation, in which ME and RE values close to zero (<0.01) and SD_R_ close to one were observed. These results corroborate those described by Andrade et al. [5], Silva et al. [16], Oliveira et al. [17], and Peixoto et al. [20], who, when studying the spatial distribution of bedding variables in CBP systems, also obtained better adjustments using the spherical and Gaussian models.

According to Ferraz et al. [37], one of the main geostatistics parameters is the nugget effect (*C*_0_), which refers to unexplained variability, considering the distance between sampled points. For all variables, the *C*_0_ values were low (near or equal to zero), an indication that they had low unexplained variability, and that the adjusted semivariograms did not have discontinuity (Table 1). Only for the pH_B-sur_ variable was the *C*_0_ value different from zero, but low, when evaluated in relation to the sill.

The *C*_0_ can be attributed to several factors, such as collection and/or analysis errors, local variations, etc. As it is not possible to quantify the contribution of each factor directly and individually, it is important that other evaluation forms are used [38]. One of the most common methods is through the spatial dependence index (SDI), which expresses the *C*_0_ in relation to the sill (*C*_0_ + *C*_1_), and makes comparisons possible, through the classification of Cambardella et al. [33]. Using the SDI to evaluate the contribution of *C*_0_ in the sill composition, it is observed that the values obtained were lower than 0.25 (Table 1). Therefore, there was strong spatial dependence for all variables, and only for pH_B-sur_ was an SDI value different from zero (0.2384) obtained, but still lower than 0.25.

According to Curi et al. [39], the results obtained with the kriging interpolation techniques are better when low nugget effect contributions are obtained for the sill. Therefore, it can be concluded that the results achieved using the kriging techniques satisfactorily represented the studied attributes. These results also show that the bedding conditions were not homogeneous in the evaluated CBP system, as stated by Andrade et al. [5].

In geostatistics, another parameter of great importance is the range (*a*), which is used to determine the spatial dependence limit, separating correlated samples from independent samples [35,40]. As can be seen in Table 1, for all the attributes, values were obtained that were greater than the shortest distance between collection points (4.5 m), with the smallest being obtained for the variable v_air,B_ (6.0831 m). The occurrence of values of a greater than the distance between collection points was an indication that the mesh used was adequate for the purposes proposed in this study.

**Figure 3:**
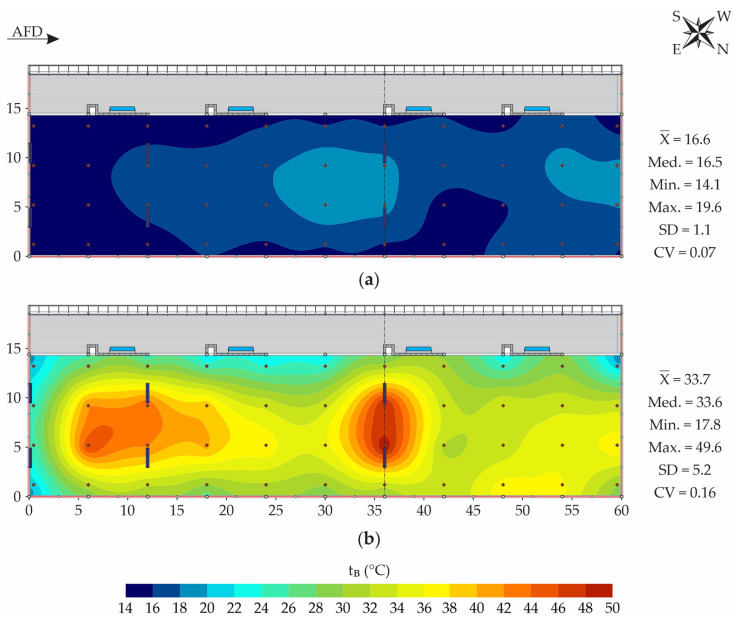
Spatial distribution of bedding temperature (t_B_, in °C) at the surface—t_B-sur_ (**a**) and at a depth of 0.2 m—t_B-20_ (**b**). AFD—air-flow direction; $\bar{X}$—mean; Med.—median; Min.—minimum; Max.—maximum; SD—standard deviation; CV—coefficient of variation.

Through the models and parameters of the semivariograms adjusted for the variables (Table 1), it was verified that they did not have random distribution in space, since there was spatial dependence occurrence. Therefore, it was possible to use ordinary kriging to predict the variables’ levels in non-sampled locations, and to obtain spatial distribution maps. Figure 3 shows the spatial distribution maps of the t_B-sur_ and t_B-20_ variables. 

Through the models and parameters of the semivariograms adjusted for the variables (Table 1), it was verified that they did not have random distribution in space, since there was spatial dependence occurrence. Therefore, it was possible to use ordinary kriging to predict the variables levels in non-sampled locations, and to get spatial distribution maps. Figure 3 shows the spatial distribution maps of the t_B-sur_ and t_B-20_ variables.

For t_B-sur_ and t_B-20_ (Figure 3), it can be observed that close mean ($\bar{X}$) and median (Med.) values were obtained, indicating that the collected data did not have accentuated asymmetry [41]. Based on the CV classification proposed by Warrick and Nielsen [27], it can be observed that the dispersion of the t_B-sur_ data was low (CV < 0.12), whereas that for the t_B-20_ data was moderate (0.12 ≤ CV < 0.24).

According to Eckelkamp et al. [8], the bedding surface temperature in CBP systems is influenced by the environment and therefore increases or decreases according to the recorded dry-bulb air temperature levels. On the bedding surface, ventilation removes heat, through the evaporation of the water part present there, and causes t_B-sur_ values to be observed close to the environment [7,42]. Considering the authors’ statement, the t_B-sur_ can be evaluated based on the dry-bulb temperature of the air (t_db_), whose hourly variation curve is illustrated in Figure 2. During the period of t_B-sur_ and t_B-20_ collection (08:00 a.m. to 09:30 a.m.), mean dry-bulb air temperature values were between 13.0 ± 2.1 °C and 19.0 ± 2.2 °C. Therefore, by recording the average t_B-sur_ values between 14.1 and 19.6 °C along the bedding area (Figure 3a), this attribute was found to be directly related to t_db_.

The t_B-sur_ results observed in this work corroborate those described by Andrade et al. [5], who evaluated a closed CBP air-conditioned system by negative pressure ventilation associated with adiabatic cooling, during the winter and summer periods, always in the morning. In the winter period, the authors reported that mean t_B-sur_ values between 13.8 and 17.8 °C were recorded, with higher levels observed in the bedding’s central region.

According to Janni et al. [11], t_B-sur_ is important for thermal comfort, given that the activity of decomposing microorganisms is non-existent or low on the bedding surface. For this reason, it is recommended that the t_B-sur_ levels be kept within the thermoneutral range for lactating dairy cattle (4.0 ≤ t_db_ < 24.0 °C, according to Nääs [43]), ensuring a thermally comfortable surface. Through Figure 3a, it is observed that the average t_B-sur_ levels recorded during the winter experimental period (08:00 a.m. to 09:30 a.m.) were lower than the upper threshold of thermal comfort for lactating dairy cattle (24.0 °C). According to Almeida et al. [44], under conditions of thermal comfort, these animals spend approximately 50.0% of the time lying down. Therefore, it can be inferred that the t_B-sur_ during the evaluated period (winter mornings) was adequate for the animals to remain lying down, a condition that makes it possible to reduce their energy expenditure to maintain their body’s core temperature.

In any case, we observed the occurrence of t_B-sur_ spatial variability along the bedding area (variation equal to 5.5 °C, Figure 3a), with lower levels recorded in the peripheral region, especially close to the southeast face of the facility (t_B-sur_ < 16.0 °C). In this region, there were two fan lines (levels 0 and 12 m, from southeast to northwest), which remained activated 24 h·day^−1^. Therefore, it can be inferred that the lowest t_B-sur_ levels occurred due to the air flow generated by the fans, which make it possible to cool the bedding surface, as stated by Black et al. [7].

On the other hand, in the central region and close to the northwest face of the bed area, higher t_B-sur_ levels were recorded (Figure 3a). It can be inferred that the higher t_B-sur_ values observed in this region are due to the low v_air,B_ recorded in these locations, which were not effective in reducing the heat on the bedding surface, as recommended by Llonch et al. [42].

Despite the fans remaining in operation 24 h·day^−1^, their distribution along the bedding area of the facility was irregular, suggesting that it was not possible to ensure homogeneous t_B-sur_ conditions (Figure 3a). Near the southeast face of the facility, four fans were installed (0 and 12 m, from southeast to northwest), but in the rest of the bedding area, there were only two more fans (36 m, from southeast to northwest). Therefore, through the results illustrated in Figure 3a, it can be inferred that the number and arrangement of fans used in this facility were not satisfactory in promoting a reduction in the average t_B-sur_ values in the entire bedding area, especially in more distant areas, such as near the northwest face of the facility.

Through Figure 3b, it can be observed that there was high t_B-20_ spatial variability along the bedding area, where mean values between 17.8 and 49.6 °C were recorded. As expected, t_B-20_ values were higher than those observed on the bedding surface. This is, therefore, an indication that the bedding decomposition process was taking place in an active manner, since the decomposing microorganisms’ activity results in heat production [7].

According to Black et al. [7], the desired temperature range in the aerobically active layer of CBP systems (0.15 to 0.30 m) corresponds to the range of 43 and 65 °C, and below 40 °C, the material degradation is slow, while, above 55 °C, pathogens are eliminated. Based on this t_B-20_ range, it was found that in a huge portion of the bedding area, the average t_B-20_ values were lower than desired in the aerobically active layer (Figure 3b).

Along the bedding area, the lowest average t_B-20_ levels were recorded in peripheral regions (southeast faces—extremity; and southwest—between the bedding and the feeding alley) (Figure 3b). Regarding the observation of low t_B-20_ values in these regions, two potential justifications can be mentioned. The first is the relative difficulty of turning over the bedding material in these places, due to the proximity to dividing walls. The second is the intense movement of the animals from the bedding area to the feeding alley, where they had access to food and water. In both cases, it is inferred that there was a trend toward the compaction of the subsurface bedding layer and, as a consequence, a reduction in the oxygen concentration, decomposition activity, and heat production was seen [45].

In contrast, mean desired t_B-20_ levels (>40 °C) were recorded only in two specific regions of the central bedding area of the facility: between the first two fans’ lines, and immediately below fan line three (Figure 3b). In the first case, it is estimated that the highest ventilation rate provided (86,000 m^3^·h^−1^ of air flow—first fans’ line) ensured the supply of the necessary aeration in the region. In the second case, it is assumed that in the region below fan line three, there was no formation of compacted subsurface structures, due to the presence of the electric dividing fence between pens 1 and 2, which prevented the animals’ movement and ensured that the bedding was not compacted. Therefore, it is understood that in this region, the supply of adequate oxygen to the decomposing microorganisms was ensured, as evidenced by the highest t_B-20_ levels recorded (Figure 3b).

**Figure 4:**
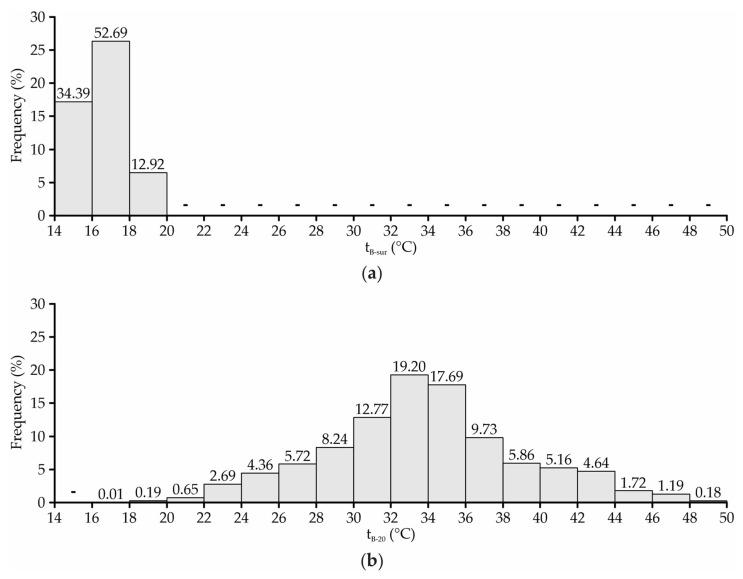
Frequency distribution of bedding temperature at the surface—t_B-sur_ (**a**) and at a depth of 0.2 m—t_B-20_ (**b**).

To obtain detailed numerical information about the fractions of area occupied by each attribute class, frequency distribution graphs of t_B-sur_ and t_B-20_ were generated, as shown in Figure 4.

In the entire bedding area, the mean t_B-sur_ values recorded were lower than 24 °C (Figure 4a), an indication that the surface temperature conditions of the bedding were favorable for the animals to remain lying down. However, given the similarity between the t_B-sur_ values and ambient temperature (t_db_) observed in this study and portrayed by other authors [5,21,42], it can be inferred that the t_B-sur_ was not suitable for the animals to remain lying down at certain times of the day. In the period from 01:00 p.m. at 04:00 p.m., t_db_ values greater than 24 °C were recorded (Figure 2). Therefore, it is estimated that in this period, the t_B-sur_ interval in some regions may have been higher than the thermal comfort threshold for lactating dairy cattle.

In approximately 87% of the bedding area, the recorded t_B-20_ was below 40 °C (Figure 4b), an indication that the bedding degradation process in this facility was occurring slowly, as well as that the compost obtained may not have had good agronomic features [17]. The t_B-20_ results portrayed in this study (Figure 4b) corroborate those observed by other authors who evaluated the bedding quality in CBP systems in Brazil and observed the occurrence of t_B-20_ values below 40 °C [5,17,21,46,47].

**Figure 5:**
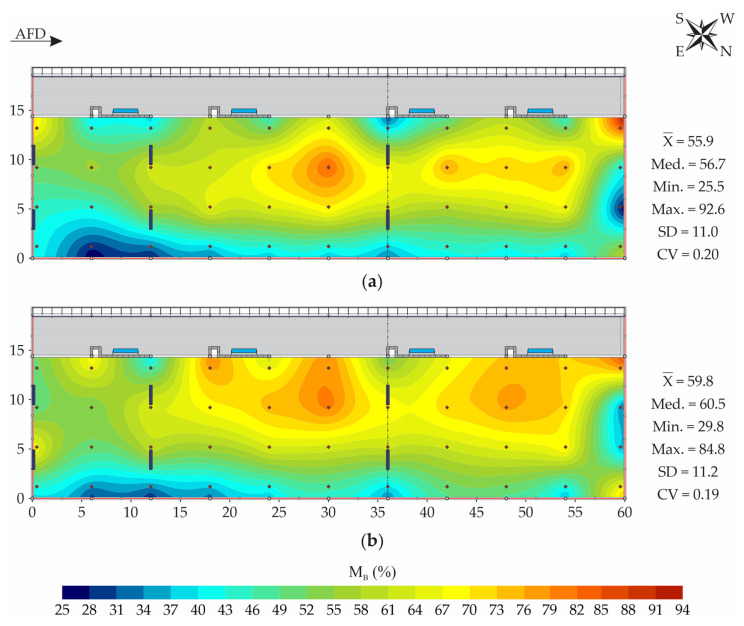
Spatial distribution of bedding moisture (M_B_, in %) at the surface—M_B-sur_ (**a**) and at a depth of 0.2 m—M_B-20_ (**b**). AFD—air-flow direction; $\bar{X}$—mean; Med.—median; Min.—minimum; Max.—maximum; SD—standard deviation; CV—coefficient of variation.

Figure 5 shows the spatial distribution maps of bedding moisture at the surface (M_B-sur_) and at a depth of 0.2 m (M_B-20_).

As verified for t_B-sur_ and t_B-20_ (Figure 3), it can be observed that for M_B-sur_ and M_B-20_, close mean ($\bar{X}$) and median (Med.) values were obtained (Figure 5). Therefore, it can be inferred that the data distributions of these variables did not have accentuated asymmetry [41]. Regarding data variability, in both cases, moderate dispersion was observed (0.12 ≤ CV < 0.24), according to the classification suggested by Warrick and Nielsen [27].

Similar spatial distributions were obtained for M_B-sur_ and M_B-20_ (Figure 5), but the variation range was greater at the bedding surface (67.1% and 55.0%, respectively). This fact occurred due to the spillage of water by the animals after ingestion and during the cleaning of the feeding alley, as well as the deposition of feces and urine in the bedding’s superficial layer. The recording of M_B-sur_ and M_B-20_ data with high variation is an indication that the ventilation system and the management applied to the bed were not able to ensure homogeneous conditions of humidity throughout the facility area.

The high variation amplitudes of M_B-sur_ and M_B-20_ depicted in this study (Figure 5) corroborate the results described by Oliveira et al. [17], who evaluated a closed CBP system during the spring period and observed variation amplitudes close to 50% at the bedding surface and at 0.2 m depth. On the other hand, they differ from the results portrayed by Andrade et al. [5], who evaluated bedding moisture in a closed CBP system during the winter period and obtained spatial distributions of M_B-sur_ and M_B-20_ with low variation amplitudes (approximately 8%).

According to Black et al. [7], the moisture levels in the bedding must be kept within the range of 40 to 60%, to ensure the maintenance of aerobic conditions and the survival of decomposing microorganisms. As one of the objectives of CBP systems is to provide a comfortable and hygienic surface for housed dairy cattle, keeping the bedding moisture below 60% is also important for the animals’ health and milk quality [4]. In the present study, however, M_B-sur_ and M_B-20_ values were greater than 60%, with higher levels always recorded near the northwest face and along the vicinity of the feeding alley (Figure 5). Close to the feeding alley, it is inferred that there was a tendency toward an increase in the bedding moisture levels, due to the spillage of water by the animals after ingestion, splashing of water during the cleaning of the feeding alley floor and greater deposition of feces and urine, as portrayed by Andrade et al. [5] and Oliveira et al. [17].

At the northwest extremity, in a region close to the feeding alley, M_B-sur_ and M_B-20_ values were always higher than 80% (Figure 5). In this location, bedding wetting occurred due to the water overflow from the feeding alley to the bedding area during floor cleaning (flushing). When cleaning the floor, there was an overload of waste and water in the gutter, causing overflow. To correct this problem, the waste gutter could be renovated and expanded, and a slightly higher dividing wall could be built at the site (>0.2 m).

Even if the greater deposition of feces and urine was observed in the region close to the feeding alley, the existing ventilation system and the bedding management should have been effective in promoting the removal and incorporation of the excess moisture in the bedding. In view of the results illustrated in Figure 5, it can be inferred that the ventilation system and bedding turning were not efficient in promoting the uniform drying of the bedding in the facility, given the elevated moisture levels observed.

**Figure 6:**
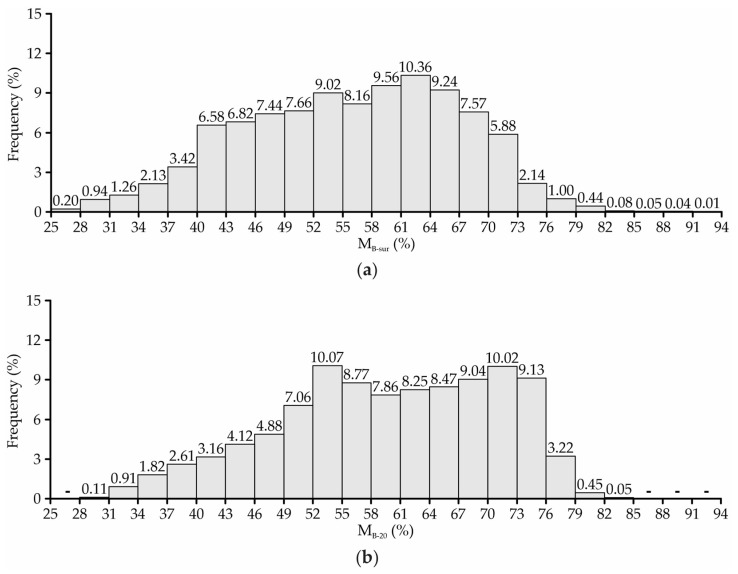
Frequency distribution of bedding moisture at the surface—M_B-sur_ (**a**) and at a depth of 0.2 m—M_B-20_ (**b**).

In the superficial layer (M_B-sur_), the bedding moisture is more important in relation to health, animal cleanliness, and milk quality. When it is excessively humid, as observed in approximately 37% of the bedding area (Figure 6a), the material tends to adhere to the surfaces of the animals’ bodies, increasing their dirtiness, mastitis risk and somatic cell count (SCC) [48].

On the other hand, in peripheral regions of the facility (northeast and northwest faces, Figure 5), M_B-sur_ and M_B-20_ values were low (<40%), which may have occurred due to excessive bedding drying in these areas, which received direct solar radiation at certain times of the day. According to Oliveira et al. [17], the registration of regions with low moisture values in the bedding surface layer is desirable, as it will provide better conditions of cleanliness and health for the animals.

Keeping M_B-20_ at adequate levels is important for the composting process. In the subsurface layer, it was observed that in approximately 5.5% of the bedding area, the M_B-20_ values recorded were lower than the minimum level recommended for the maintenance of the bedding composting process (40%) (Figure 6b). Although not very representative, it can be inferred that the decomposition activity was slower or nonexistent in this bedding area percentage, since M_B-20_ levels below 40% can lead to a reduction in the populations of decomposing microorganisms [1,49].

According to Damasceno [1], when the bedding material is excessively humid (>60%), as observed in around 49% of the bedding subsurface area (Figure 6b), the spaces available for oxygen circulation are filled by water. Consequently, there is a reduction in the oxygen concentration, causing an anaerobiosis condition. Therefore, considering the t_B-20_ and M_B-20_ values recorded in the bedding’s subsurface layer (Figure 4b and Figure 6b), it is estimated that the material decomposition process occurred slowly in this facility. Furthermore, based on the low t_B-20_ levels and high M_B-20_ levels recorded in the bed, it can be inferred that the compost formed may not have good agronomic features, due to the potential nutrient leaching and non-elimination of pathogens [50,51].

The high M_B-20_ values portrayed in this study (Figure 6b) differ from those reported by other authors [5,17,47,52,53]. These authors evaluated the bedding moisture levels in the subsurface layer (0.15–0.20 m) of CBP systems in distinct locations in Brazil and observed that the M_B-20_ values were always lower than 70%.

Regarding bedding management in this CBP system, two points must be considered. The first is related to the bedding’s replacement with dry material, which was performed twice during the period, but the amount added was not sufficient to maintain the bedding moisture at adequate levels. The second refers to the turning of the bedding twice a day (09:00 a.m. and 4:00 p.m.), with a long interval between the second turning of one day and the first of the next day (16–18 h). Based on the M_B-sur_ and M_B-20_ results obtained in this study (Figure 5 and Figure 6), which reflected the bedding conditions after an extended period without turning, it is pertinent to consider performing one additional daily turning operation, preferably at the time of the first milking (04:00 a.m.). At the same time, it is recommended to reallocate the times of the other turnings to late morning (after 11:00 a.m.) and late afternoon (after 5:00 p.m.). In this way, it is expected that it will be possible to promote the homogenization of the material, incorporation of oxygen, and removal of excess moisture levels, considering that the bedding’s adequate management is a primary factor for the CBP system’s performance [1,4,48].

**Figure 7:**
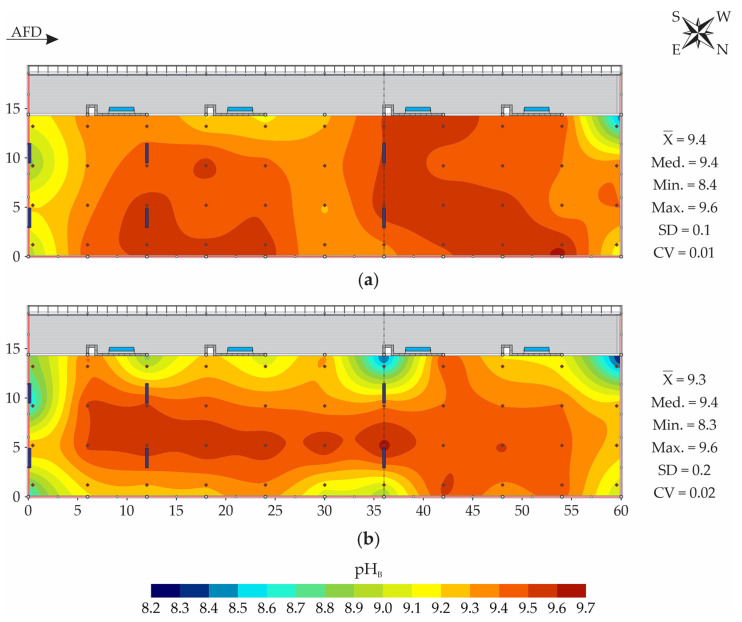
Spatial distribution of bedding hydrogenionic potential (pH_B_) at the surface—pH_B-sur_ (**a**) and at a depth of 0.2 m—pH_B-20_ (**b**). AFD—air-flow direction; $\bar{X}$—mean; Med.—median; Min.—minimum; Max.—maximum; SD—standard deviation; CV—coefficient of variation.

Figure 7 illustrates the spatial distribution maps of pH_B-sur_ and pH_B-20_ during the winter period. The mean and median values of pH_B-sur_ and pH_B-20_ were close and, therefore, their distributions did not mark asymmetry [41]. In both cases, the CV was less than 0.12 and characterized by low variability, according to the classification proposed by Warrick and Nielsen [27].

In both bedding layers evaluated, the pH showed low spatial variability during the winter period (Figure 7), with variation amplitudes equal to 1.2 and 1.3, at the surface and at 0.2 m depth, respectively. The spatial distribution behaviors were similar on the surface and subsurface, an indication that the phenomena that govern the distribution of this variable in the bedding are similar between layers, and that the bedding turning ensured the relative homogenization of the material along the profile. Considering that the bedding decomposition process occurred in an analogous manner along the profile, it is pertinent to state that the compost generated at the end of the composting process may have slightly uniform agronomic features along the bedding depth, as portrayed by Oliveira et al. [17].

**Figure 8:**
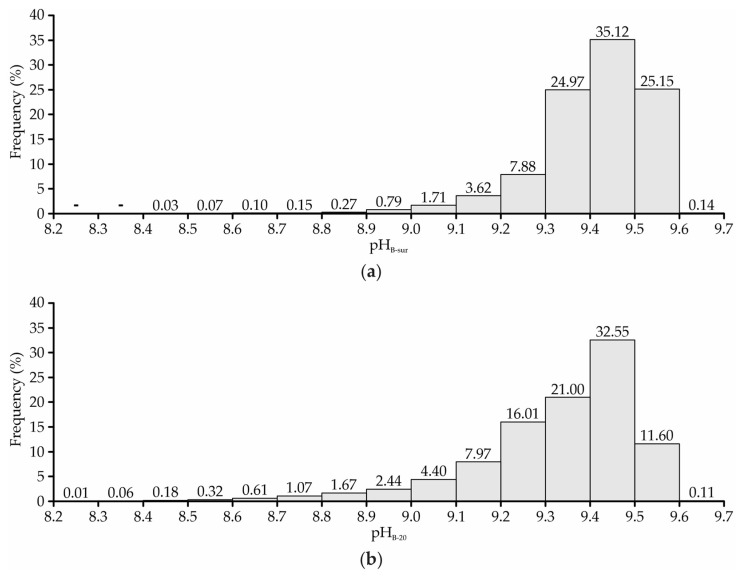
Frequency distribution of bedding hydrogenionic potential at the surface—pH_B-sur_ (**a**) and at a depth of 0.2 m—pH_B-20_ (**b**).

According to Pereira Neto [54], the pH range considered ideal for most decomposing microorganisms is between 5.5 and 8.0. Other authors claim that the optimal range is wider, given that most enzymes are active in the range of 5.5 to 8.5 and, therefore, this would be the desired range during the composting process [55]. In the present study, it was observed that the bedding pH values were above the recommended values in the entire bedding area, in both evaluated layers (surface and 0.2 m deep, Figure 7). pH values lower than 8.5 were obtained only in some peripheral regions of the bedding area (on the four faces). By means of joint spatial distribution map observations of temperature (Figure 3), humidity (Figure 5), and bedding pH (Figure 7), it was verified that the regions in which the lowest average pH values were obtained (≤8.5) coincided with the locations where lower temperatures (<30 °C) and/or higher moisture levels (>80%) were observed. Therefore, we can infer that the lower pH levels in these places were due to slow or nonexistent decomposition activity, due to the low temperatures and high moisture recorded in the bedding material, which can limit or inhibit the development of microorganisms acting in the composting process [7,49]. On the other hand, pH values lower than 8.5 were not very representative in the system evaluated in this study, given that they were recorded in less than 1% of the bedding area (Figure 8).

The average levels of pH_B-sur_ and pH_B-20_ were mostly higher than 8.5 (Figure 7 and Figure 8). Results such as those observed are common in advanced stages of the composting process, in which the complete consumption of the organic acids formed in the initial stage has already occurred, or when the carbon:nitrogen ratio (C:N) is low [56]. The high pH values obtained are also evidence that there may have been losses of nitrogen (N) by volatilization, which are particularly high when the pH is greater than 7.0, according to Bernal et al. [57].

The bedding pH results portrayed in this study (Figure 7 and Figure 8) corroborate those observed by other authors, in CPB systems in distinct locations and times of the year. Andrade et al. [5] evaluated the bedding pH spatial distribution in a closed CBP system during the winter period and observed the occurrence of values greater than 8.7. Oliveira et al. [17] evaluated the bedding pH in a closed CBP system and obtained pH spatial distributions with values mostly greater than 9.0. Oliveira et al. [47] evaluated CBP systems in the south of the state of Minas Gerais and described the occurrence of mean pH_B-20_ values of 9.0 ± 0.8. Fávero et al. [52] carried out studies on CBP systems in the state of São Paulo and observed the occurrence of pH_B-20_ values greater than 8.5 in the winter period. Radavelli et al. [53] carried out a characterization study of CBP systems in the west of Santa Catarina state and described the occurrence of mean pH_B-20_ values of 8.7 ± 0.5.

According to Fávero et al. [51], pH levels greater than 8.8 may inhibit the growth of environmental mastitis pathogens. Therefore, it can be inferred that the high pH levels observed in the present study (Figure 7 and Figure 8) may have been beneficial for the animals’ health in the evaluated CBP system, as well as for the milk quality produced.

In CBP systems, the bedding composting process generally occurs within a year, and it may be accelerated or delayed depending on the material used as a substrate, the animal stocking, the management employed and the frequency and volume of bedding replacement with dry material. The high pH_B-sur_ and pH_B-20_ values (Figure 7 and Figure 8) observed in this study may be an indication that the C:N ratio of the bedding material present in this facility was low, despite this material having been changed only four months before the study started. With the intense decomposing microorganisms’ activity, common in the initial composting stage, the carbon initially made available is usually consumed, making it necessary to initiate new additions via replacement with dry material. It is through bedding replacement that an adequate carbon supply is ensured, and pH values are maintained within the recommended range for decomposing microorganisms. As the frequency and amount of dry bedding added by replenishment at this facility was low, the pH levels were above the recommended values, and the bedding already showed low carbon availability. These conditions led to low decomposition activity and low heat production, as already portrayed through the t_B-20_ spatial distribution maps (Figure 3). In cases such as this, it is recommended that more bedding replacement operations be conducted with dry material, as well as that the volume added in each replacement be greater.

**Figure 9:**
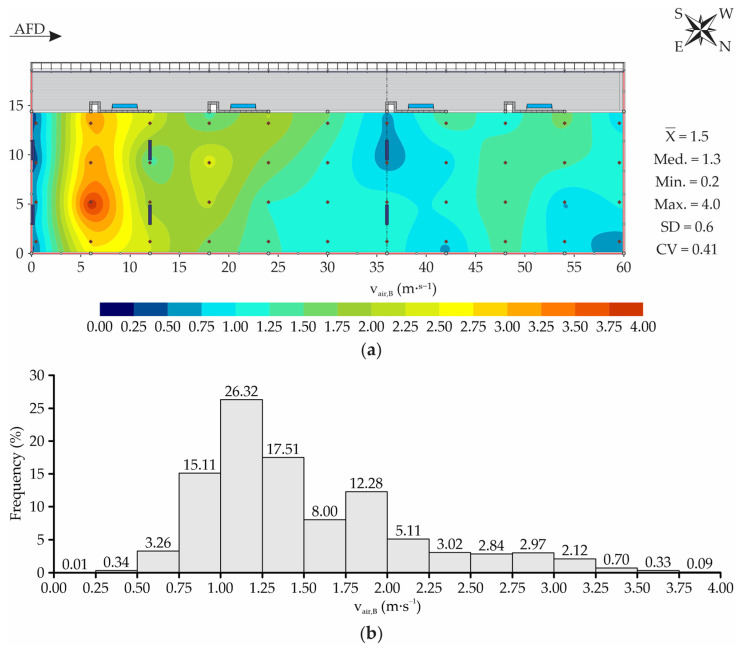
Spatial (**a**) and frequency (**b**) distribution of air velocity at bedding level (v_air,B_). AFD—air-flow direction; $\bar{X}$—mean; Med.—median; Min.—minimum; Max.—maximum; SD—standard deviation; CV—coefficient of variation.

The spatial distribution map and frequency distribution graph of the air velocity at bedding level (v_air,B_) are illustrated in Figure 9. Through the figure, it can be observed that the spatial distribution of v_air,B_ was highly heterogeneous throughout the bedding area, given that average values between 0.2 and 4.0 m∙s^−1^ were recorded (variation equal to 3.8 m∙s^−1^).

For CBP systems, it is recommended that the air velocity be kept close to 1.8 m∙s^−1^ throughout the animal-occupied zone (AOZ), to ensure bedding drying, removing gases, and favoring thermal exchanges [7,11]. Considering 1.8 m∙s^−1^ as the minimum air velocity for cooling and bedding drying (MVCBD), it was found that in the bedding area, there were regions with v_air,B_ values lower than desired (Figure 9a). Along the bedding area, the largest v_air,B_ magnitudes were recorded in the zones where the first two fans’ lines operated, an indication that they were due to air currents generated by mechanical ventilation. However, we also noted the occurrence of zones with low v_air,B_ in the regions below fan lines one and three, and in the northwest extremity of the facility.

Fagundes et al. [58] evaluated the airflow generated by mechanical fans with low volume and high rotation (LVHS) using computational fluid dynamics (CFD) techniques. The authors observed that in the region immediately below the first fan line, a zone with low air velocity occurred. Of course, as in the CBP facility studied, the fans were installed at a 3.0 m height and with 45° inclination in relation to the horizontal; the airflow in the immediately anterior region was sucked in and directed by the equipment, forming an area with low v_air,B_ below the first ventilation line (Figure 9a).

In this study, the v_air,B_ spatial distribution along the bedding area is an indication that the number and arrangement of mechanical fans present in the facility was not adequate, given that regions with low v_air,B_ also formed in the central and posterior regions of the bedding area. Regarding the central and posterior regions of the facility, it can be inferred that the low v_air,B_ values recorded were due to the distance being greater than that recommended between fan lines (12.0 m, according to Damasceno [1]). As the distances between the second and third ventilation lines and the northwest end were 24.0 m, it was observed that the existing ventilation system was not sufficient to ensure homogeneous v_air,B_ conditions along the entire bedding area (Figure 9a). Only in the first 20.0 m of the bedding area, from the southeast to northwest of the facility, the mean v_air,B_ values were equal to or higher than that recommended for drying the bed, removing gases, and favoring thermal exchange (1.8 m∙s^−1^). In the rest of the area, as well as in the southeast extremity (near the taller wall), the v_air,B_ magnitude was lower than MVCBD.

The v_air,B_ results portrayed in this study (Figure 9) corroborate those observed by Oliveira et al. [59], who evaluated the spatial distribution of thermal conditions in open CBP systems and with different ventilation types (natural, mechanical HVLS and mechanical LVHS) during the fall period. In the CBP system with LVHS ventilation, the authors observed that the v_air,B_ distribution was not uniform, and that there was a predominance of v_air,B_ magnitudes below 1.8 m∙s^−1^ in 58.4% of the bedding area. In the present study, it was observed that v_air,B_ was lower than recommended in approximately 70% of the bedding area (Figure 9b). Through this observation, the inference is reinforced that the number and distribution of LVHS fans along the evaluated system did not meet the need for ventilation required at the site. To improve the air velocity uniformity throughout the bedding area and, consequently, the levels of temperature and bedding moisture surface, it is recommended that the ventilation system used in the place be resized, changing the number and arrangement of fans present.

## 4. Conclusions

Through the application of geostatistical techniques, it was possible to verify that the variables of bedding temperature at the surface (t_B-sur_) and at a depth of 0.2 m (t_B-20_), bedding moisture at the surface (M_B-sur_) and at a depth of 0.2 m (M_B-20_), bedding hydrogenionic potential at the surface (pH_B-sur_) and at a depth of 0.2 m (pH_B-20_), and air velocity at bedding level (v_air,B_) had spatial dependence. For all these variables, the occurrence of strong spatial dependence was verified, enabling the application of kriging interpolation techniques and the generation of spatial distribution maps.

From the generated maps, it was possible to observe that the variables t_B-sur_, t_B-20_, M_B-sur_, M_B-20_, and v_air,B_ showed high spatial variability along the bedding area of the facility, while the attributes pH_B-sur_ and pH_B-20_ had low variation (8.2–9.7). In the superficial layer, the bedding temperature was below 20 °C (14.1 to 19.6 °C), indicating that it was comfortable for the animals to remain lying down. However, the average M_B-sur_ values recorded in the region close to the feeding alley were higher than 60.0%, a condition that can generate health and milk quality problems. At 0.2 m depth, t_B-20_ levels predominantly below 40.0 °C (87.0% of the area) and M_B-20_ above 60.0% (49.0% of the area) were observed in some local areas (peripheral regions and close to the feeding alley), indications that the bedding material’s decomposition process was taking place slowly, as reflected by the high pH_B-sur_ and pH_B-20_ values (>9.0). Regarding v_air,B_, a non-uniform distribution and velocity magnitudes lower than 1.8 m∙s^−1^ were found in approximately 70.0% of the bedding area of the facility. Adequate v_air,B_, levels were observed only in the first 20 m of the facility, from southeast to northwest, because of the fan lines present in this region.

## Figures and Tables

**Table 1:** Methods, models, and parameters estimated from the semivariograms adjusted for the mean values of bedding temperature at the surface (t_B-sur_) and at the depth of 0.2 m (t_B-20_), bedding moisture at the surface (M_B-sur_) and at the depth of 0.2 m (M_B-20_), bedding pH at the surface (pH_B-sur_) and at the depth of 0.2 m (pH_B-20_) and air velocity at bedding level (v_air,B_), during the experimental winter period.

Variable	Method	Model	*C*_0_	*C*_1_	*C*_0_ + *C*_1_	*a*	*a′*	SDI	ME	SD_M_	RE	SD_R_
t_B-sur_	REML	Gaussian	0.0000	2.8010	2.8010	6.6460	11.5038	0.0000	−0.0774	0.8309	−0.0479	1.0218
t_B-20_	REML	Gaussian	0.0000	76.6540	76.6540	6.4740	11.2061	0.0000	−0.4229	4.3183	−0.0477	0.9759
M_B-sur_	REML	Spherical	0.0000	274.2200	274.2200	10.2400	10.2387	0.0000	−0.2836	14.1767	−0.0104	0.9928
M_B-20_	REML	Spherical	0.0000	248.1420	248.1420	9.9570	9.9571	0.0000	−0.1750	13.8022	−0.0066	1.0018
pH_B-sur_	REML	Spherical	0.0165	0.0527	0.0692	27.5256	27.5256	0.2384	−0.0053	0.2053	−0.0139	1.0618
pH_B-20_	REML	Spherical	0.0000	0.1079	0.1079	8.6834	8.6834	0.0000	−0.0078	0.3130	−0.0130	1.0251
v_air,B_	REML	Gaussian	0.0000	0.6892	0.6892	6.0831	10.5288	0.0000	−0.0120	0.4273	−0.0116	0.8959

*C*_0_—nugget effect; *C*_1_—contribution; *C*_0_ + *C*_1_—sill; *a*—range; *a′*—practical range; SDI—spatial dependence index; ME—mean error; SD_M_—mean-error standard deviation; RE—reduced error; SD_R_—reduced-error standard deviation; and REML—restricted maximum likelihood.

## Data Availability

The data presented in this study are available upon request from the corresponding author.
